# Enhancing Deep Learning–Based Subabdominal MR Image Segmentation During Rectal Cancer Treatment: Exploiting Multiscale Feature Pyramid Network and Bidirectional Cross-Attention Mechanism

**DOI:** 10.1155/ijbi/7560099

**Published:** 2025-07-23

**Authors:** Yu Xiao, Xin Yang, Sijuan Huang, Lihua Guo

**Affiliations:** ^1^School of Electronic and Information Engineering, South China University of Technology, Guangzhou, Guangdong Province, China; ^2^State Key Laboratory of Oncology in South China, Guangdong Key Laboratory of Nasopharyngeal Carcinoma Diagnosis and Therapy, Guangdong Provincial Clinical Research Center for Cancer, Sun Yat-sen University Cancer Center, Guangzhou, Guangdong, China

**Keywords:** bidirectional cross-attention, biomedical image segmentation, multiscale feature pyramid network, subabdominal MR image segmentation, U-Net

## Abstract

**Background:** This study is aimed at solving the misalignment and semantic gap caused by multiple convolutional and pooling operations in U-Net while segmenting subabdominal MR images during rectal cancer treatment.

**Methods:** We propose a new approach for MR Image Segmentation based on a multiscale feature pyramid network and a bidirectional cross-attention mechanism. Our approach comprises two innovative modules: (1) We use dilated convolution and a multiscale feature pyramid network in the encoding phase to mitigate the semantic gap, and (2) we implement a bidirectional cross-attention mechanism to preserve spatial information in U-Net and reduce misalignment.

**Results:** Experimental results on a subabdominal MR image dataset demonstrate that our proposed method outperforms existing methods.

**Conclusion:** A multiscale feature pyramid network effectively reduces the semantic gap, and the bidirectional cross-attention mechanism facilitates feature alignment between the encoding and decoding stages.

## 1. Introduction

In recent years, the integration of magnetic resonance (MR) imaging into radiation treatment planning and therapy workflows has gained significant traction. This innovative approach, known as MR-guided adaptive radiotherapy, effectively capitalizes on MR's superior soft-tissue contrast [1–3]. While the abdomen presents specific challenges—primarily due to the presence of multiple organs-at-risk (OARs) and the complexities of contouring them—these obstacles can be overcome. The traditional manual contouring of OARs on abdominal MR images is not only labor-intensive but also introduces variability among different observers [4, 5]. However, the implementation of computer-aided automated multiorgan segmentation stands out as a highly effective solution to enhance accuracy and efficiency [6–8].

Ronneberger et al. [9] established the U-Net architecture for biomedical image segmentation. U-Net is a powerful architecture that enables the effective reuse of features through jump connections, effectively addressing the challenge of maintaining spatial relationships. However, there are notable issues to consider. First, decoding features typically exhibit some degree of information loss compared to encoding features, resulting in a semantic gap that can impact performance. Additionally, after numerous convolutional and pooling operations, a misalignment can occur in the spatial location and distribution of features. This issue is particularly critical in the segmentation of subabdominal MR images, where even seasoned specialists may find it challenging to distinguish between the anal canal and rectum. The essential difference between these two structures lies in the transition along the internal surface from endodermal to skin-like ectodermal tissue, a distinction that demands considerable clinical expertise. Without proper alignment of the spatial relationships of the organs, the performance can significantly degrade or even fail entirely in the segmentation process.

The issue of spatial misalignment in U-Net architectures has been previously identified in studies such as [10, 11]. Zhou et al. [10] highlighted that the substantial differences between shallow and deep convolutional layers lead to semantic inconsistency and spatial misalignment between encoder and decoder feature maps. Directly concatenating these features via skip-connections introduces noisy shallow features into the output, compromising discriminability and leading to misclassifications. To address this, Li et al. [11] developed a multiscale diffractive U-Net for improved multiscale feature fusion, while Zhou et al. [10] introduced a semantic consistency enhancement module based on attention mechanisms. However, their approach primarily reinforces important features or channels through similarity computations without explicitly resolving spatial misalignment.

In contrast, we argue that explicit spatial alignment calibration is essential. To this end, we propose a bidirectional cross-attention mechanism (BiCA) designed to preserve spatial information in U-Net and mitigate misalignment. Additionally, given the proven efficacy of feature pyramid networks (FPNs) in tasks such as image classification, object detection, and semantic segmentation, we integrate FPN into our framework for subabdominal MRI segmentation. This integration is aimed at bridging the semantic gap inherent in traditional U-Net approaches, ultimately enhancing segmentation accuracy. Based on these considerations, this paper tries to address the challenges of organ and tumor segmentation in subabdominal MR images through the introduction of two innovative modules. The first module seamlessly integrates U-Net with a multiscale feature pyramid network (MFP-Net), effectively bridging the semantic gap. MFP-Net extends the capabilities of the FPN [12], utilizing a multiscale structure to generate robust pyramid features within a convolutional neural network. The second module introduces a powerful BiCA, encompassing both channel and spatial attention strategies to maintain spatial integrity and eliminate misalignment. This approach significantly enhances comprehensive representation and markedly improves the accuracy of automatic organ segmentation. A preprint of this groundbreaking work has already been published [13] in Arxiv.

This article presents our significant contributions to the field:

1. We have developed MFP-Net, an innovative model specifically engineered for subabdominal MR image segmentation. MFP-Net is equipped with advanced features such as dilated convolutions, feature reuse, and a multiscale feature pyramid architecture. Its primary goal is to enhance the capture of spatial relationship information, effectively bridging the semantic gap between encoding and decoding.
2. We have introduced a robust BiCA that seamlessly integrates both channel attention and spatial attention. This mechanism ensures the preservation of spatial relationships and minimizes misalignment between the encoding and decoding stages.
3. We have conducted comprehensive experiments using our proposed model on a subabdominal MR image dataset from the Sun Yat-sen University Cancer Center. Our method demonstrates remarkable improvements in segmentation accuracy compared to other prevalent medical image segmentation techniques.

## 2. Related Work

U-Net [9] was a symmetrical neural network with an encoder–decoder structure and skip connections. The encoder used some convolutional and downsampling layers to extract depth features, while the decoder received semantic information from the bottom of the “U” and restored spatial information through skip connections. Inspired by U-Net, Ozan et al. [14] introduced an attention gate to U-Net and developed Attention U-Net. Isensee et al. [15] focused on the universality of the U-Net model and made minimal modifications by changing the activation function and regularization strategy while systematizing the segmentation task to adapt to multiple datasets. Huang et al. [16] proposed a SE-connection pyramid network (SECP-Net), which designed a SE-connection module and a pyramid structure for improving the segmentation performance. Additionally, with vision transformers gaining attention in the field of computer vision, TransUNet [17] combined U-Net and transformer to become the first medical image segmentation model using transformer, while Swin-unet [18] was born in combination with Swin transformer block. Since Transformers are hampered by quadratic computational complexity, Valanarasu et al. [19] introduced visual state space blocks as basic blocks to capture a wide range of contextual information with fewer convolutional layers. The success of U-Net and its variants has demonstrated their potential for advancing medical image segmentation models.

In the field of subabdominal image segmentation, particularly for female pelvic cavity images, privacy concerns arise due to the high cost associated with acquiring medical data. Currently, there are no publicly available datasets specific to the female pelvic cavity. Li et al. [20] used a small amount of data from six institutions and proposed a prototype network-based segmentation model to perform segmentation in eight regions of interest (ROI) in the male pelvic cavity, including the bladder, rectum, and seminal vesicles. Balagopal et al. [21] employed cascaded 2D and 3D U-shaped networks to segment the prostate, bladder, rectum, and femoral head in pelvic cavity CT images. Their approach began with five 2D networks to approximate the organ locations, followed by five 3D networks for precise determination of the organ boundaries. While this method was effective, it was also computationally demanding and time-consuming. Lei et al. [22] combined CT and MRI for multiorgan segmentation in the male pelvis cavity, and Rundo et al. [23] fused the squeeze-and-excitation (SE) attention module into U-Net to segment endangered organs of the male pelvis in MRI images from multiple institutions. Zhang et al. [12] proposed another method that was based on U-Net, employing a multiscale residual structure to capture distant contextual information for segmenting multiple organs in the pelvis of men with prostate cancer. To summarize, the field of subabdominal image segmentation encounters significant challenges, including limited datasets and the practical clinical difficulties in distinguishing between certain organs, such as the anal canal and rectum.

## 3. Methods

### 3.1. MR Data

All experiments were conducted with the approval of local institutional review boards. Eighty-three patients (including 23 patients with rectal cancer) were recruited with informed consent and scanned using 3T MR scanners (MAGNETOM Vida; Siemens) with an 18-channel surface coil and an integrated spine coil. Real-time 2D images using a spoiled GRE sequence (coronal orientation, TR/TE = 3.6/1.3 ms, FA = 9°, *resolution* = 1.6 × 1.6 *mm*^2^, slice thickness = 6 mm, temporal resolution = 300 ms, and scan time = 1 min) were acquired. The MR data of all patients were provided by the esteemed Sun Yat-sen University Cancer Center. Their age, gender, and other information are shown in Table 1. The majority of these patients are men over 40 years old. All personally identifiable information was meticulously removed to safeguard privacy. For effective label visualization, we employed the Nibabel library in Python to parse the raw data, which was in medical DICOM format. We focused on five primary organs in the pelvic region for segmentation: the anal canal, bladder, rectum, left femoral head, and right femoral head. Our manual segmentation process established a robust ground truth, with a human expert—typically a physician—meticulously segmenting and labeling images slice by slice, especially within the context of 3D volumetric imagery. This manual approach is recognized as the most precise method, as accurately identifying structures in medical images presents significant challenges. A trained operator thoroughly reviews around 80 images to delineate the contours of the target structures with precision. To ensure high reliability in labeling these five organs, two gastroenterology surgeons and one imaging surgeon used photoshop graphics software for the manual labeling process. This labeling was rigorously verified by a gastroenterology surgery specialist with over 20 years of experience. The labeled data was then categorized into training (*n* = 55; 66%), validation (*n* = 9; 11%), and testing (*n* = 19; 23%) sets, with image slices cropped to a matrix size of 256 × 256, focusing on the central region containing all relevant organ structures. To further enhance the dataset, we implemented advanced in-flight data augmentation techniques, including random rotations between [−5°, 5°], random contrast adjustments, elastic transformations, and random horizontal flips. Additionally, we performed image normalization to minimize skewness, ensuring a robust and reliable dataset for our study.

### 3.2. The Framework of Our Method

**Figure 1:**
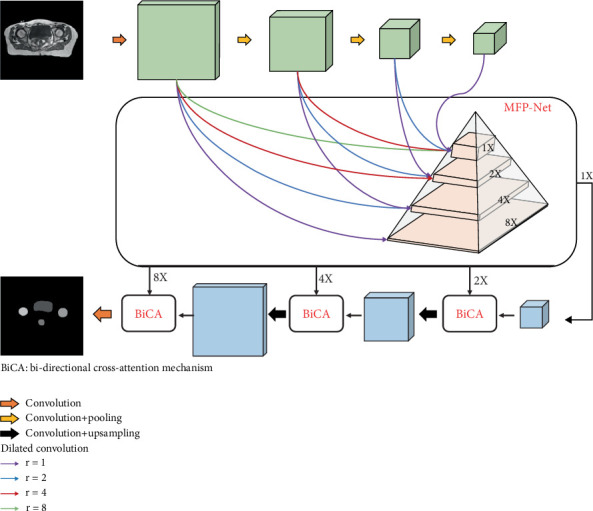
The main framework of our method.

Our approach employs two distinct modules: a MFP-Net and a BiCA, as illustrated in Figure 1. The MFP-Net consists of reused features, dilated convolutions, and a multiscale feature pyramid architecture. Central to the network is the pyramid structure, where we apply varying dilation rates in dilated convolutions to multiplex encoding features across multiple scales. The features derived from the same scale are then concatenated to enhance the network's feature representation capabilities. As we progressively integrate more feature multiplexing modules into the decoding process, the network increasingly refines its ability to capture spatial relationships, thereby optimizing the decoding outcome. For further technical details, please refer to Section 3.3. However, the MFP-Net architecture may experience misalignment issues between encoding and decoding due to the series of convolutional and pooling operations. To address this challenge, we implement a BiCA during decoding. This mechanism combines channel attention, which aligns channel features between decoding and encoding, with spatial attention, which harmonizes the spatial features between the two processes. For additional technical details, please consult Section 3.4.

### 3.3. MFP-Net

MFP-net utilizes a multiscale structure to construct multiscale pyramid features within U-Net. Assuming *X*_*E*_ and *X*_*D*_ represent the feature mapping in encoding and decoding in U-Net, respectively, *X*_*F*_ denotes the feature mapping of the intermediate process. Given input *I* ∈ *R*^1×*H*×*W*^, the convolutional neural network obtains features *X*_*E*_^*i*^ at different scales by encoding and pooling for *i* ∈ (1, 2, ⋯*n*) times, where *X*_*E*_^*i*^ ∈ *R*^*C*_*i*_×*H*/2^*i*−1^×*W*/2^*i*−1^^. For each *X*_*E*_^*i*^, the following operations are performed:

(1) $$\begin{array}{c} X_{F}^{ik}=D_{k}\left(H\left(X_{E}^{i-1}\right)\right)\;k\leq n-i, \end{array}$$

where *D*_*k*_(∙) denotes a dilated convolution with a dilation rate  *k* and *H*(∙) denotes a single encoding operation. *X*_*F*_^ik^  is a set of new features at the pooling time *i* and dilation rate *k*. The input to the decoding network is *X*_*D*_^1^ = [*X*_*F*_^*i*1^]. The decoding operation can be expressed as follows:

(2) $$\begin{array}{c} X_{D}^{i}=U\left(H\left(X_{D}^{\text{i}-1}\right)\right)+X_{F}^{\text{ik}}, \end{array}$$

where *U*(∙) denotes upsampling and *X*_*F*_^ik^ are features from the encoding network, having the same number of channels and resolution as *U*(*H*(*X*_*D*_^*i*−1^)). These improvements leverage the reuse module, and they are helpful for gradually recovering spatial information. The pyramid structure can extract features of different scales using various dilation rates. It also involves extracting features of the same scale through dilated convolution with different dilation rates. These features are then transmitted to decoding at different scales. From another perspective, the reused structure facilitates the fusion of features at different scales explicitly, and the decoding network chooses effective information that is more useful for decoding and spatial recovery.

### 3.4. A BiCA

The BiCA comprises two parts: the channel attention mechanism and the spatial attention mechanism. They are described separately as follows:

• Channel attention mechanism

**Figure 2:**
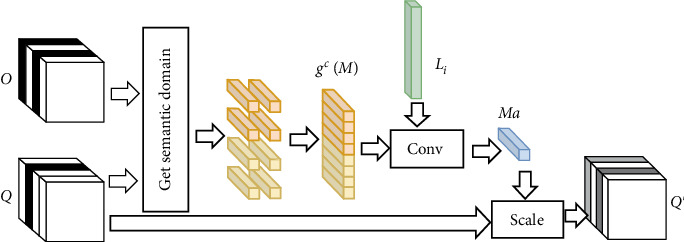
The main framework of the channel attention mechanism based on semantic domain adaptation.

As shown in Figure 2, the channel attention is computed through two input sources, which are encoding features *O* ∈ *R*^*C*×*H*×*W*^ generated in pyramid network and output *Q* ∈ *R*^*C*×*H*×*W*^ in decoding networks, respectively. These two sources are divided into blocks with *H*_0_ × *W*_0_  size; then, calculate their means in each block as follows:

(3) $$\begin{array}{c} S_{d}^{c}\left(X\right)=\frac{1}{H_{0}\times W_{0}}\overset{\left(d+1\right)H_{0}}{\underset{i=dH_{0}}{\sum }}\overset{\left(d+1\right)W_{0}}{\underset{j=dW_{0}}{\sum }}X_{\text{ij}}^{c}. \end{array}$$

*S*_*d*_^*c*^(*X*) from two input sources are flattened into one dimension as *g*^*c*^(*O*) and *g*^*c*^(*Q*) with (*H*/*H*_0_ × *W*/*W*_0_) × *C* size and concatenate them together,

(4) $$\begin{array}{c} g^{c}\left(M\right)=\left[g^{c}\left(O\right),g^{c}\left(Q\right)\right]\in R^{\left(2\times H/H_{0}\times W/W_{0}\right)\times C\times 1}. \end{array}$$

The mask *Ma* can be obtained by learning the channel weight *L*_*i*_ as follows:

(5) $$\begin{array}{c} Ma={\left[\overset{2\times H/H_{0}\times W/W_{0}}{\underset{i=1}{\sum }}L_{i}g_{i}^{c}\left(M\right)\right]}_{c}. \end{array}$$

The module of “Get Semantic Domain” is to calculate the mean of each block by Equation (5), and then, they are flattened and combined as *g*^*c*^(*M*) by Equation (6). The mask *Ma* is obtained using 1∗1 convolution of the channel weight *L* and *g*^*c*^(*M*) by Equation (7). The output of the attention mechanism *Q*′ is obtained by the dot product of *Ma* and output of decoding network *Q*, which can be considered as scaling processing. Therefore, *Q*′ is consistent with *O* and *Q* in channel dimension, and it can be a plug-and-play module of the U-Net.

• Spatial attention mechanism

The spatial attention mechanism is used for calibrating spatial bias. The calibration is done by calculating the bias between the encoding and decoding features. This bias is then used to calibrate the encoding features to make them consistent with the decoding features. Semantic flow [24] represents the offset of semantic information between two feature maps. In our method, we borrowed it to calculate the spatial bias, and then calibrated the bias by weighting the neighbors. The process of calculating the bias is explained in detail below.

**Figure 3:**
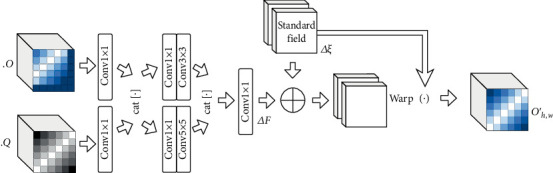
The main framework of the spatial attention mechanism using semantic flow calibration.

As shown in Figure 3, features *O* ∈ *R*^*C*×*H*×*W*^ from encoding and features *Q* ∈ *R*^*C*×*H*×*W*^ from decoding are, respectively, downsampling by 1∗1 convolution, then concatenated together, and parallelly input into two modules with a series of convolutional transformations. After a series of convolutional transformations, the output Δ*F* ∈ *R*^*H*×*W*×2^ is finally downscaled into two channels, and each channel represents a directional offset. The input features *O* are calibrated by the following steps:

(6) $$\begin{array}{c} \varphi \left(O\right)=G\left(O,\Delta F+\Delta \xi \right), \end{array}$$

where Δ*ξ* ∈ *R*^*H*×*W*×2^ is the standard offset field whose pixel takes values on a range of (−1, −1) ~ (1, 1). *G* represents the deformation warp operation, which adjusts input *O* to new space according to offset mapping. Warp operation has two steps. Firstly, the positional correspondence of mapping is obtained,

(7) $$\begin{array}{c} G_{h^{\prime }}=\left\{\begin{array}{cc} \min\left(1,\Delta F_{h}+\Delta \xi_{h}\right), & \Delta F_{h}+\Delta \xi_{h}>0,\\ \max\left(-1,\Delta F_{h}+\Delta \xi_{h}\right), & \Delta F_{h}+\Delta \xi_{h}<0, \end{array}\right.\\ G_{w^{\prime }}=\left\{\begin{array}{cc} \min\left(1,\Delta F_{w}+\Delta \xi_{w}\right), & \Delta F_{w}+\Delta \xi_{w}>0,\\ \max\left(-1,\Delta F_{w}+\Delta \xi_{w}\right), & \Delta F_{w}+\Delta \xi_{w}<0. \end{array}\right. \end{array}$$

Input *O*(*h*, *w*) is adjusted as *O*(*h* + *G*_*h*′_, *w* + *G*_*w*′_). Secondly, the new feature *O*_*h*,*w*_′ is obtained by the bilinear interpolation,

(8) $$\begin{array}{c} O_{h,w}^{\prime }=\underset{p\in \tilde{N}}{\sum }L_{p}O\left(h+G_{h^{\prime }},w+G_{w^{\prime }}\right) \end{array}$$

where $\tilde{N}$ represent four neighbor points of *p* and the weight *L*_*p*_ of each neighbor is assigned by the distance between the neighbor point and the point *p*.

• BiCA

**Figure 4:**
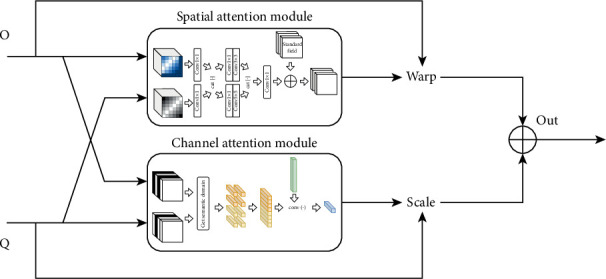
The main framework of the BiCA.

The attention mechanisms discussed above share two key characteristics. Firstly, both mechanisms have two input sources and produce a single output result. Secondly, each mechanism calibrates just one feature channel: The encoding feature (denoted as “O”) is adjusted through wrapping, while the decoding feature (denoted as “Q”) is refined through scaling. When combined, these two attention mechanisms can form a bidirectional cross-attention network, as illustrated in Figure 4. This configuration allows the attention mechanism to produce dual outputs, utilizing information from both input sources. In this approach, features generated by the feature pyramid pass through the channel attention module, while features from the decoding layer are processed via the spatial attention module. The resulting features are then aggregated point by point to serve as input for the subsequent decoding unit.

The attention mechanisms discussed above share two key characteristics. Firstly, both mechanisms have two input sources and produce a single output result. Secondly, each mechanism calibrates just one feature channel: The encoding feature (denoted as “O”) is adjusted through wrapping, while the decoding feature (denoted as “Q”) is refined through scaling. When combined, these two attention mechanisms can form a bidirectional cross-attention network, as illustrated in Figure 4. This configuration allows the attention mechanism to produce dual outputs, utilizing information from both input sources. In this approach, features generated by the feature pyramid pass through the channel attention module, while features from the decoding layer are processed via the spatial attention module. The resulting features are then aggregated point by point to serve as input for the subsequent decoding unit.

## 4. Results

### 4.1. Experimental Settings

#### 4.1.1. Hardware and Software Configuration

The experiments were conducted on an NVIDIA RTX 2080 Ti GPU (11 GB VRAM) using Python 3.8 and PyTorch 1.9.0. The model leverages CUDA 11.1 and NVIDIA cuDNN for parallel computing acceleration.

#### 4.1.2. Training Protocol

The network was trained with a batch size of 32 for 400 epochs. The initial learning rate was set to 0.01 and decayed to 0.001 and 0.0001 at the 200th and 300th epochs, respectively. Optimization was performed using stochastic gradient descent (SGD) with a momentum of 0.9 and a weight decay of 0.001.

#### 4.1.3. Validation and Testing

A five-fold cross-validation strategy was applied to 55 cases. The best-performing model on the validation set was selected for final evaluation on a held-out test set of 19 cases, ensuring no overlap with training data.

#### 4.1.4. Others

In medical image segmentation tasks, 3D deep networks are generally regarded as more effective than their 2D counterparts. However, due to our GPU's memory capacity of 11 GB, we were constrained to set the batch size to 1 to prevent memory overflow. This limitation resulted in the 2D deep networks outperforming our chosen classical 3D medical image segmentation method, H-DenseUNet [2]. One contributing factor is that a smaller batch size typically leads to diminished performance in deep networks. Furthermore, in a 2D network, each slice is treated as an individual training sample, whereas in a 3D network, all slices from a single patient are combined into a single training sample. Consequently, the 2D deep network had access to a larger training set compared to the 3D network. Considering these factors, we opted to utilize 2D networks for our experiments.

### 4.2. Evaluation Metrics

Two evaluation metrics are utilized to assess the model's performance: the three-dimensional Dice coefficient (also known as the volumetric Dice coefficient (DSC)) and the mean surface distance (MSD) [25].

The DSC is used to measure the degree of overlap between two samples and takes values in the range [0, 1]. The larger the DSC is, the more similar the two samples are. The formula calculating DSC is as follows:

(9) $$\begin{array}{c} \text{DSC}=\frac{2\left|A⋂B\right|}{\left|A⋃B\right|}, \end{array}$$

where *A* and *B* are automated and manual 3D segmentations of the organs, respectively. MSD is calculated as follows:

(10) $$\begin{array}{c} \text{MSD}\left(A,B\right)=\frac{1}{\left|S\left(A\right)+S\left(B\right)\right|}\left(\underset{a\in S\left(A\right)}{\sum }d\left(s_{A},S\left(B\right)\right)+\underset{b\in S\left(B\right)}{\sum }d\left(s_{B},S\left(A\right)\right)\right), \end{array}$$

where *S*(*A*) is the set of surface points in *A* and *S*(*B*) is the set of surface points in *B*, $d\left(v,S\left(A\right)\right)=\underset{s_{A}\in S\left(A\right)}{\min}\left\|v-s_{A}\right\|$. That is the minimum value of Euclidean distance from this point to all points of another object.

### 4.3. Analysis of Experimental Results

In our experiments, we employed five-fold cross-validation for both the ablation and contrast studies. Each experiment was run 10 times, and we calculated the mean and standard deviation of the metrics. Additionally, a paired sample *t*-test was used as the statistical method for conducting post hoc tests on the experimental results.

We initially selected the popular U-Net [9] model as our comparative algorithm. However, we also incorporated a U-Net with point-by-point addition (U-Net-Add) as a baseline method, since it uses point-by-point addition instead of the concatenation operation found in the MFP-net structure. For comparison, we included several attention-based methods of U-Net, such as UNet++ [26] and r2u-net [27], as well as a transformer structure known as MedT [19].

To distinguish between the methods, we named the model that employs only dilated convolution as MFP-Net1, while the model that combines dilated convolution with multiscale feature pyramid structures is referred to as MFP-Net2. To ensure a fair comparison among the different methods, we considered the model size. We varied the number of channels in the initial convolution operation, testing configurations with 8, 16, and 32 channels, which correspond to different U-Net structures with varying parameter counts. The results of these experiments are presented in Tables 2 and 3.

Tables 2 and 3 demonstrate that MedT utilizes the transformer structure for medical image segmentation. It is important to mention that MedT shows minimal improvement on this task and requires over five times the training time, with a slower fitting speed and greater memory requirements. Its performance on the Dice coefficient is subpar, but its performance on MSD is not the lowest. This is due to the transformer structure's capacity to capture contextual dependencies, which is particularly advantageous for MSD, as it is sensitive to outlier sample points.

We performed a paired sample *t*-test with a 95% confidence level to determine the significance of the differences in the average Dice and MSD obtained from our MFP-Net and five other medical image segmentation methods. The results, including the average improvement and significance, are presented in Table 4. Our MFP-Net demonstrated a statistically significant improvement in both the average Dice and MSD, with *p* < 0.05, confirming the validity of our statistical analysis.

**Figure 5:**
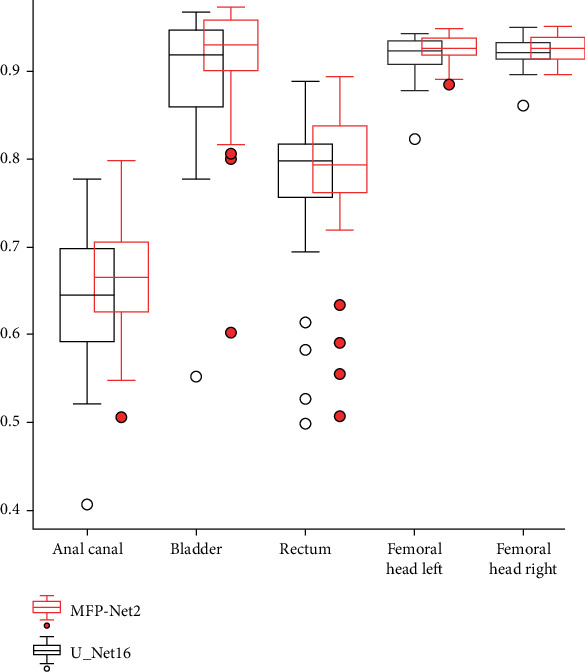
Comparison of the Dice coefficient box-lines of MFP-Net2 and U-Net.

Specifically, for each case, box-line plots are used to show the distribution characteristics of statistical data, as shown in Figure 5. Comparing MFP-Net2 with U_Net16, the boxes of the five organs' segmentation accuracies are higher using MFP-Net2 than using U_Net16, and the outlier points are closer to the box using MFP-Net2 than using U_Net16. The results show that MFP-Net2 achieves better performance than U_Net16.

We conducted a comparison of the Dice scores obtained from our MFP-Net and U-Net16 on five organs. To investigate the significance of the differences, we performed a paired sample *t*-test with a 95% confidence level and presented the Dice improvement and significance in Table 5. Notably, our MFP-Net achieved a 2.9% improvement in Dice for the anal canal organ, which requires significant clinical experience to distinguish. This improvement was statistically significant (*p* < 0.05), further validating the efficacy of our approach.

According to the analysis above, it can be concluded that MFP-Net can improve the segmentation performance of organs while using fewer parameters. To compare the segmentation results of different methods, representative methods are visualized separately in three dimensions from various perspectives. It was observed that some methods had isolated prediction points that deviated from the organs, increasing the false positive values and affecting their DSC. The more distant the isolated points were, the larger the MSD, supporting the conclusion that MFP-Net can improve the segmentation performance of organs. This conclusion is consistent with the results in Tables 2 and 3 and Figure 5.

MedT is insensitive to training boundaries and can achieve similar performance when determining whether an organ is present in the slice based on the overall image information, thus avoiding the generation of false positives. It is worth noting that the jaggedness of the segmentation boundary is clear in MedT's visual images due to the limitation of video memory, as the experiment reduced the size of input images during the training. However, the huge memory requirements of the transformer structure made it necessary to abandon this learning framework.

In this study, we adopt MFP-Net as our backbone architecture and perform a comprehensive comparison with several state-of-the-art attention mechanisms, including SE [23], gather-excite (GE) [28], convolutional block attention module (CBAM) [29], and attention U-Net [14]. To enhance the performance of MFP-Net, we systematically integrate three representative attention structures—SE (channel attention), GE (spatial attention), and CBAM (hybrid channel-spatial attention)—into the skip connections using a unified framework. Additionally, we propose an attention block with dual-channel input capability and incorporate it into the U-Net architecture, resulting in an improved variant termed Attention U-Net. To validate the efficacy of our proposed BiCA, we conduct extensive experiments under controlled conditions. The results demonstrate significant improvements in performance metrics compared to baseline methods.

It is evident from Table 6 that the proposed method, which utilizes a BiCA, achieved better results for each organ in the MFP network framework, compared to the initial MFP network. The combination of MFP and BiCA resulted in the best Dice coefficient values at the anal canal, rectum, femoral head (left), and femoral head (right), outperforming other attention methods. In particular, the segmentation of the anal canal, which is a small organ with ambiguous boundaries, was found to be challenging. BiCA was proposed to address the issue of ambiguous boundaries caused by semantic ambiguity, which is more pronounced in smaller organs. In conclusion, the BiCA is an effective solution to the feature mismatch problem caused by multiresolution feature aggregation and can solve the ambiguity generated in jumping connections, thereby improving the ability to accurately segment regional boundaries.

**Figure 6:**
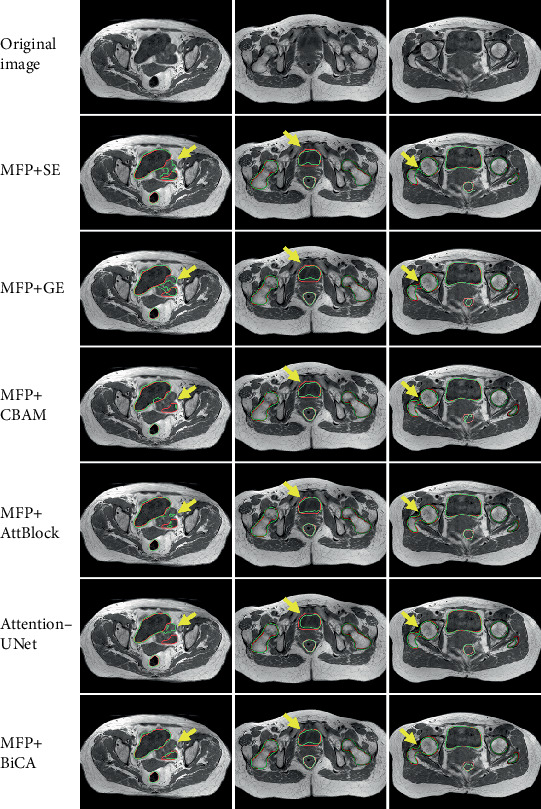
Slice segmentation results. The red contour is the label, and the green contour is the prediction of different methods. The yellow arrows point to the areas where the difference in error is more significant.

We compared the performance of our proposed method to existing attention mechanisms by outlining contours in 2D and visually comparing the results.  Figure 6 illustrates that our method outperforms the others in accurately delineating organ edge contours. This superior performance is due to the BiCA module's ability to differentially discriminate feature maps, which aligns with the findings presented in Table 4. It is worth noting that while 3D visualization can provide an overview of prediction performance, this comparison in 2D outlines allows for a more detailed analysis.

### 4.4. Functional Visualization

**Figure 7:**
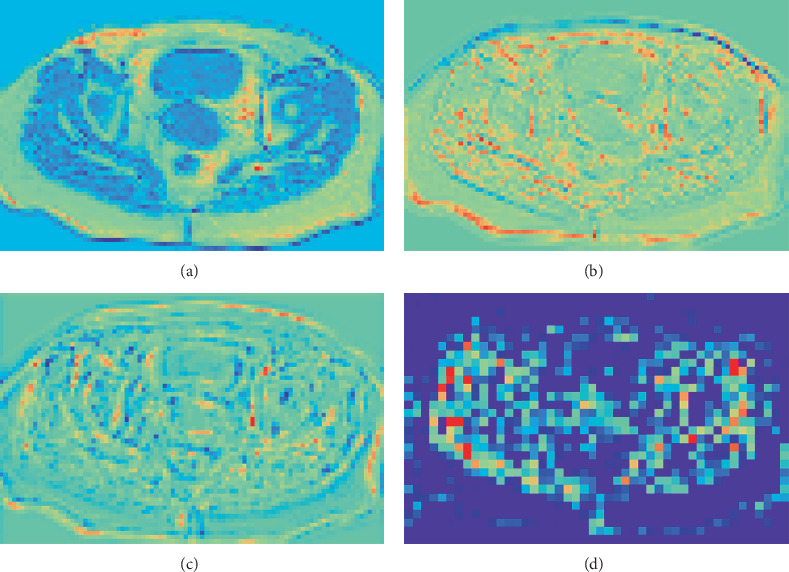
Feature heat maps: (a) after low dilation rate, (b) after middle dilation rate, (c) after high dilation rate, and (d) the decoded heat map from the decoder.

To demonstrate the functionality of the multiscale features pyramid network, we utilize heat maps generated from the dilated convolution layer to provide visual explanations. These feature heat maps are computed using gradient class activation mapping (Grad-CAM) during the training phase. By employing dilated convolution for spatial position alignment, all four maps maintain the same resolution. Figures 7a, 7b, and 7c display the heat maps obtained from dilated convolutions with varying dilation rates, while Figure 7d showcases the heat map generated by the corresponding decoding network. A comparison reveals that the image boundary in Figure 7d appears increasingly blurred relative to Figures 7a, 7b, and 7c, indicating that the decoding process has lost significant spatial information. In this study, to ensure the effective recovery of spatial relationship information during decoding, features derived from dilated convolutions are jointly transmitted to the decoding network. This approach facilitates the extraction of both spatial relationships and spatial alignment within a single decoding process. Consequently, our multiscale features pyramid network integrates dilated convolutions and a multiscale feature pyramid structure to capture spatial relationship information, thereby addressing the semantic gap between encoding and decoding.

**Figure 8:**
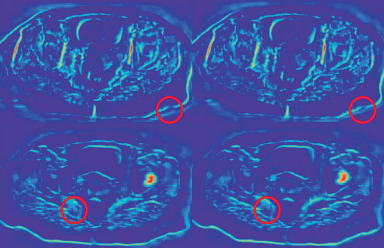
Comparison of feature heat maps. Left column: direct summation; right column: BiCA. Red circles in the first row point out the jagged problem, and red circles in the second row point out the alignment problem.

To demonstrate the functionality of the BiCA, feature heat maps are calculated to compare with and without BiCA, as shown in Figure 8. The left column contains feature maps that are directly summed without BiCA, and the right column contains feature maps that are summed after BiCA. Features in the right column are less jagged due to the neighborhood interpolation algorithm in the spatial attention mechanism, which is highlighted by red circles in the first row. The features of the left are not well aligned compared to those of the right, which are highlighted by red circles in the second row.

**Figure 9:**
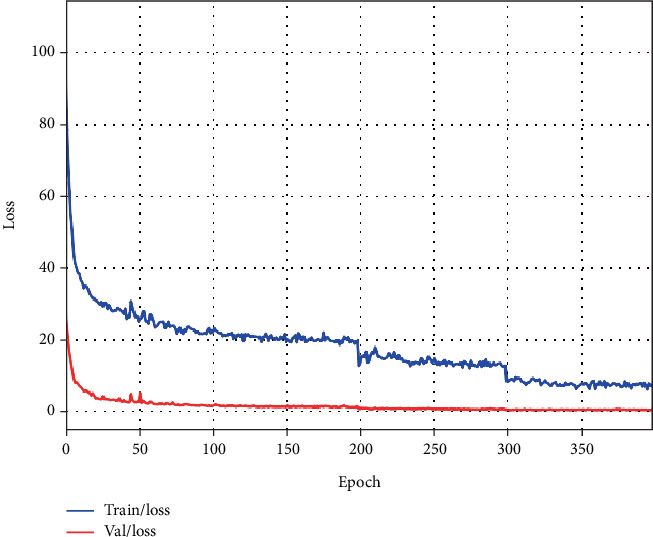
The loss curves with epochs during the training and validation.

To evaluate potential overfitting, we present the training and validation loss curves in Figure 9. The parallel convergence trends observed in both curves demonstrate that our method maintains strong generalization capability without exhibiting overfitting behavior.

## 5. Discussion

We have developed an innovative MRI image segmentation approach that uses a MFP-Net and a BiCA, demonstrating strong performance. While 3D networks offer detailed geometric context, they are more prone to overfitting and demand more memory and computational resources. Our multiscale 2D network serves as an effective alternative.

Recent advances have introduced several memory-efficient 3D segmentation approaches [30, 31] for medical imaging. In future work, we plan to integrate these memory optimization techniques with our method, potentially creating an optimal solution that balances accuracy with computational efficiency.

Our study has several limitations that warrant further investigation. First, the dataset was relatively small, with only 19 test cases, necessitating validation on larger and more diverse cohorts to ensure generalizability. Semisupervised learning methods [32] and modality adaption methods [33, 34] can be considered as the effect solution to solve the limitation of a small dataset Second, ground truth annotations were generated through a single reader's initial labeling followed by senior reader refinement, which precludes assessment of inter- and intra-observer variability. Future work should incorporate multiple expert readers to establish consensus contours and improve annotation reliability. Third, while our method enhances segmentation performance for the anal canal and rectum, the achieved Dice scores (below 80%) remain insufficient for clinical adoption. Further optimizations are essential to ensure the accuracy and robustness required for real-world medical applications. Finally, while our model has demonstrated superior performance on abdominal MRI datasets from Sun Yat-sen University Cancer Center, its generalizability across diverse clinical settings remains to be validated. In real-world applications, MRI data may exhibit significant heterogeneity due to variations in scanning equipment, protocols, and institutional practices. Therefore, extending the proposed method's applicability to multicenter datasets will constitute a critical direction for our future research. Moreover, beyond conventional 3D approaches, an important research direction worth exploring involves integrating self-supervised pretraining techniques with segmentation tasks. This strategy would leverage large-scale, nonannotated datasets to learn robust feature representations through pretraining, followed by task-specific fine-tuning for medical image segmentation. Such a paradigm could potentially enhance model performance while reducing dependency on annotated medical data.

## 6. Conclusions

This paper presents a comprehensive analysis of the traditional encoder-decoder network structure's limitations, particularly its inability to effectively convey robust spatial relationship information during the decoding process due to the inadequacy of jump connections. To overcome these constraints, the study introduces two novel strategies: (1) The integration of a U-Net-based structure with dilated convolution and MFP-Nets during the encoding phase and the reuse of multiscale features during decoding. This approach helps mitigate the semantic gap by efficiently leveraging the wide receptive field of dilated convolution for sequential extraction of new features. These new features are then fused to provide the decoding network with more robust spatial information. In the decoding phase, the MFP-Net enhances spatial information recovery by merging corresponding reused features before each decoding. (2) The implementation of a dual attention mechanism that preserves spatial information while minimizing misalignment issues. This mechanism helps rectify channel and spatial discrepancies between the encoding and decoding processes, facilitating spatial alignment prior to their integration.

## Figures and Tables

**Table 1:** Relevant statistical information of the patient.

	**Age**		**Gender**		**Rectal cancer**	
**Range**	**> 40**	**≤ 40**	**Famale**	**Male**	**True**	**False**
Number of patients	56	27	35	48	23	60

**Table 2:** Number of parameters and Dice coefficients under different network structures (mean ± SD).

**Network structure**	**Model size (M)**	**Anal canal**	**Bladder**	**Rectum**	**Femoral head (left)**	**Femoral head (right)**	**Average**
UNet-8 [9]	2.07	0.628 ± 0.085	0.888 ± 0.085	0.761 ± 0.092	0.903 ± 0.034	0.908 ± 0.030	0.8176
UNet-16 [9]	8.25	0.639 ± 0.083	0.894 ± 0.085	0.766 ± 0.099	0.916 ± 0.026	0.919 ± 0.018	0.8268
UNet-32 [9]	32.95	0.652 ± 0.076	0.891 ± 0.096	0.775 ± 0.102	0.920 ± 0.022	0.920 ± 0.019	0.8316
UNet++ [26]	8.75	0.641 ± 0.084	0.904 ± 0.082	0.781 ± 0.108	0.923 ± 0.017	0.925 ± 0.017	0.8348
r2u-net [27]	24.35	0.622 ± 0.081	0.895 ± 0.082	0.731 ± 0.118	0.912 ± 0.018	0.921 ± 0.015	0.8162
MedT [19]	88.59	0.549 ± 0.088	0.843 ± 0.089	0.687 ± 0.111	0.869 ± 0.030	0.867 ± 0.027	0.7828
UNet-Add [9]	6.67	0.642 ± 0.077	0.902 ± 0.082	0.740 ± 0.117	0.910 ± 0.014	0.920 ± 0.015	0.8228
MFP-Net1	8.09	0.670 ± 0.076	0.899 ± 0.088	0.763 ± 0.112	0.923 ± 0.021	0.927 ± 0.014	0.8364
MFP-Net2	8.07	0.668 ± 0.071	0.905 ± 0.079	0.773 ± 0.096	0.924 ± 0.016	0.924 ± 0.015	**0.8388**

*Note:* The best results are shown in bold.

**Table 3:** Model size and MSD for different network structures (in millimeters).

**Network structure**	**Model size (M)**	**Anal canal**	**Bladder**	**Rectum**	**Femoral head (left)**	**Femoral head (right)**	**Average**
UNet-8 [9]	2.07	2.416 ± 1.812	2.652 ± 4.437	1.921 ± 1.809	0.870 ± 1.009	0.469 ± 0.290	1.6656
UNet-16 [9]	8.25	1.742 ± 1.274	2.064 ± 3.744	1.342 ± 1.426	0.425 ± 0.161	0.418 ± 0.132	1.1982
UNet-32 [9]	32.95	3.403 ± 7.479	2.264 ± 4.017	1.674 ± 1.454	0.407 ± 0.210	0.437 ± 0.197	1.6370
UNet++ [26]	8.75	2.875 ± 5.529	1.194 ± 1.471	1.568 ± 1.540	0.435 ± 0.356	0.322 ± 0.074	1.2788
r2u-net [27]	24.35	1.995 ± 2.565	2.911 ± 4.906	5.797 ± 6.888	5.035 ± 7.519	1.266 ± 2.458	3.4008
MedT [19]	88.59	1.878 ± 0.682	1.273 ± 0.632	2.332 ± 2.045	0.650 ± 0.219	0.322 ± 0.074	1.291
UNet-Add [9]	6.67	1.191 ± 0.432	2.296 ± 3.973	3.486 ± 5.230	1.902 ± 2.599	0.662 ± 0.499	1.9074
MFP-Net1	8.09	1.639 ± 1.271	1.795 ± 1.703	3.308 ± 4.310	0.420 ± 0.321	0.393 ± 0.225	1.5110
MFP-Net2	8.07	1.608 ± 1.261	1.167 ± 1.685	1.556 ± 1.393	0.373 ± 0.122	0.357 ± 0.115	**1.0122**

*Note:* The best results are shown in bold.

**Table 4:** Average improvement and significance of our MFP-Net versus five segmentation methods.

**Network structure**	**Average improvement of DICE (%)**	**The significance of improvement**	**Average improvement of MSD (mm)**	**The significance of improvement**
Vs U-Net16	1.2	*p* < 0.05	0.186	*p* < 0.05
Vs r2u-net	0.4	*p* < 0.05	0.2666	*p* < 0.05
VS UNet++	2.26	*p* < 0.05	2.3886	*p* < 0.05
Vs MedT	5.6	*p* < 0.05	0.2788	*p* < 0.05
Vs UNet-Add	1.6	*p* < 0.05	0.8952	*p* < 0.05

**Table 5:** Dice improvement and significance of our MFP-Net versus U-Net16 on five organs.

	**Anal canal**	**Bladder**	**Rectum**	**Femoral head left**	**Femoral head right**
Dice improvement of MFP-Net (%)	2.9	1.1	0.7	0.8	0.5
The significance of improvement	*p* < 0.05	*p* < 0.05	*p* > 0.05	*p* > 0.05	*p* > 0.05

**Table 6:** Segmentation Dice coefficients for different methods (mean ± SD).

**Methods**	**Anal canal**	**Bladder**	**Rectum**	**Femoral head (left)**	**Femoral head (right)**	**Average**
MFP	0.668 ± 0.071	0.905 ± 0.079	0.773 ± 0.096	0.924 ± 0.016	0.924 ± 0.015	0.8388
MFP + SE	0.663 ± 0.077	0.903 ± 0.085	0.772 ± 0.098	0.925 ± 0.018	0.924 ± 0.016	0.8374
MFP + GE	0.653 ± 0.079	0.900 ± 0.085	0.762 ± 0.108	0.921 ± 0.025	0.922 ± 0.015	0.8361
MFP + CBAM	0.669 ± 0.077	0.912 ± 0.073	0.773 ± 0.110	0.921 ± 0.022	0.926 ± 0.017	0.8402
MFP + AttBlock	0.665 ± 0.080	0.908 ± 0.085	0.781 ± 0.102	0.925 ± 0.016	0.926 ± 0.017	0.8410
AttentionUNet	0.642 ± 0.085	0.908 ± 0.085	0.781 ± 0.102	0.925 ± 0.016	0.926 ± 0.017	0.8410
MFP + BiCA	0.678 ± 0.075	0.906 ± 0.084	0.785 ± 0.095	0.928 ± 0.019	0.929 ± 0.015	**0.8452**

*Note:* The best results are shown in bold.

## Data Availability

The private MR image dataset is unavailable due to privacy or ethical restrictions. All of the code is available for production or analysis during this investigation. Any further inquiries should be forwarded to the corresponding author.
